# Comparative effects of resistance- and assistance-based robot training on brain activation and motor recovery in stroke patients

**DOI:** 10.3389/fneur.2026.1756167

**Published:** 2026-03-06

**Authors:** Il-Ho Kwon, Won-Seob Shin

**Affiliations:** 1 Department of Physical Therapy, Graduate School of Daejeon University, Daejeon, Republic of Korea; 2 Department of Rehabilitation & Assistive Technology, National Rehabilitation Center, Ministry of Health and Welfare, Seoul, Republic of Korea; 3 Department of Physical Therapy, College of Health Medical Science, Daejeon University, Daejeon, Republic of Korea

**Keywords:** assistance based robot training, functional near-infrared spectroscopy (fNIRS), prefrontal cortex, resistance based robot training, stroke

## Abstract

**Background:**

Stroke is a significant cause of disability worldwide, often resulting in persistent upper-limb dysfunction. Robot-assisted therapy has emerged as an effective rehabilitation strategy by enabling intensive, repetitive, and task-specific training. In this study, we aimed to investigate the effects of resistance- versus assistance-based robotic interventions on brain activation and motor recovery in patients with stroke, as these effects remain insufficiently understood.

**Methods:**

Twenty-five adults with hemiparetic stroke were randomized to a resistance-based robot training group (RTG, *n* = 13) or assistance-based robot training group (ATG, *n* = 12). Interventions were delivered using the InMotion 2.0 for 30 min per session, five sessions/week over 4 weeks. Brain activation was measured using functional near-infrared spectroscopy (fNIRS), motor function using the Fugl–Meyer Assessment for the Upper Limb (FM-UL) and kinematic indices from InMotion 2.0, and activities of daily living using the Motor Activity Log (MAL).

**Results:**

Prefrontal activation decreased from pre- to post-intervention in both groups. In the ipsilesional hemispheres, differences between groups were significant (*p* < 0.05). In the resistance training group, additional improvements were found in mean velocity, circle size, and movement independence (*p* < 0.05). Both groups showed significant gains in FM-UL and ADL performance (*p* < 0.05), with no significant between-group differences in these measures.

**Conclusion:**

Resistance-based robotic training was associated with greater motor improvements in kinematic smoothness, and larger reductions in prefrontal activation within ipsilesional hemispheres compared with assistance-based training. These findings suggest differences in prefrontal activation patterns accompanied by improvements in kinematic movement smoothness in stroke survivors.

**Clinical trial registration:**

https://cris.nih.go.kr, (Registration Number: KCT0011076).

## 1 Introduction

Stroke is a leading cause of disability and motor impairment, particularly prevalent among the growing elderly population, that affects more than 1 million individuals annually in the European Union and over 700,000 in the United States, accounting for 2–4% of total healthcare expenditures (1–4). Stroke can be classified as an ischemic or hemorrhagic disorder that disrupts cerebral blood supply. In ischemic stroke, blood flow to the brain is obstructed by thrombosis, whereas in hemorrhagic stroke, it is interrupted by internal bleeding. As a result, cortical tissue is partially destroyed, leading to impaired neural commands in the sensorimotor cortex, reduced or absent selective muscle activation, and ultimately, reduced arm and hand function (3, 5). Upper-limb recovery is particularly challenging due to its anatomical complexity, and brain regions responsible for upper-limb motor control are especially vulnerable to stroke damage (5). According to Rossini et al. (6), the degree of recovery depends largely on the lesion location and its severity. Approximately 60% of stroke survivors continue to experience upper-limb impairment 6 months after stroke onset (7). Moreover, more than 60% of stroke survivors exhibit persistent neurological deficits, resulting in limitations in activities of daily living (ADL) and cognitive impairments such as aphasia and hemispatial neglect, while 30–66% are unable to functionally use their affected arm (3, 8).

Constraint-induced movement therapy (CIMT), a widely adopted rehabilitation strategy, has been shown to promote cortical reorganization by combining intensive, repetitive training of the affected limb with restraint of the unaffected limb. This approach not only yields functional benefits but also enhances patient motivation and increases the use of the paretic arm in daily activities (9–12). Robot-assisted therapy also provides quantitative measurements and supports objective monitoring of rehabilitation progress. Most robotic devices incorporate ADL-based training, as functional and task-oriented interventions have demonstrated favorable outcomes in stroke rehabilitation (13, 14). Therapy emphasizing ADL is also referred to as a motor relearning program. Several studies have reported that robot-assisted therapy produces greater improvements in motor outcomes compared with conventional approaches (15–17).

The extent of motor recovery after stroke varies considerably depending on lesion type, location, and size. Although the precise relationship between neuroplasticity and functional recovery remains to be fully understood, neuroimaging studies using PET and fMRI have demonstrated cortical reorganization in patients with partial or complete upper-limb recovery (18–20). These findings highlight involvement not only of the cerebral hemispheres but also of other cortical areas, such as the premotor cortex, supplementary motor area, and parietal cortex, suggesting that recovery is mediated by a distributed neural network (21–23). Functional near-infrared spectroscopy (fNIRS) is a non-invasive optical technique that enables evaluation of brain activity by measuring cerebral oxygenation and hemodynamic responses (24). fNIRS detects changes in oxygenated hemoglobin (oxy-Hb), deoxygenated hemoglobin (deoxy-Hb), and total hemoglobin. Oxy-Hb concentration serves as an indicator of local cerebral blood flow (CBF) (25), and fluctuations in oxy-Hb correspond to cortical activation levels comparable to those observed in fMRI studies (26).

Selecting a rehabilitation modality tailored to a patient’s upper-limb functional status can significantly enhance the effectiveness of robot-assisted therapy. Therefore, identifying the most appropriate training mode is a key factor in optimizing rehabilitation outcomes. In robotic rehabilitation, various training modes—such as passive, active-assistive, active, and resistive—are commonly employed to meet the diverse needs of patients with different levels of motor impairment (27). Previous systematic reviews (28) highlighted the need to investigate the neurophysiological mechanisms underlying optimal training in stroke patients—particularly those associated with resistive training—and to determine which training modalities are most suitable for individuals with stroke.

Resistance-based robotic training requires active force generation against external loads, thereby increasing movement-related effort and cognitive demand compared with assistance-based training. Tasks involving greater cognitive–motor integration and effort have been shown to preferentially engage the prefrontal cortex (PFC), which plays a key role in attention, motor planning, and executive control during complex motor tasks (29–33). Based on this framework, we hypothesized that, compared with assistance-based training, resistance-based training would lead to greater pre–post reductions in task-evoked ipsilesional PFC O_2_Hb (fNIRS) and greater improvements in upper-limb kinematic smoothness (UL-KD).

To date, few studies have directly compared brain activation during robot-assisted therapy delivered in resistance versus assistance modes in stroke patients. Therefore, the present study aimed to investigate the effects of robot-assisted therapy on brain activation, upper-limb function, and activities of daily living in individuals with stroke.

## 2 Materials and methods

### 2.1 Study design

**Figure 1:**
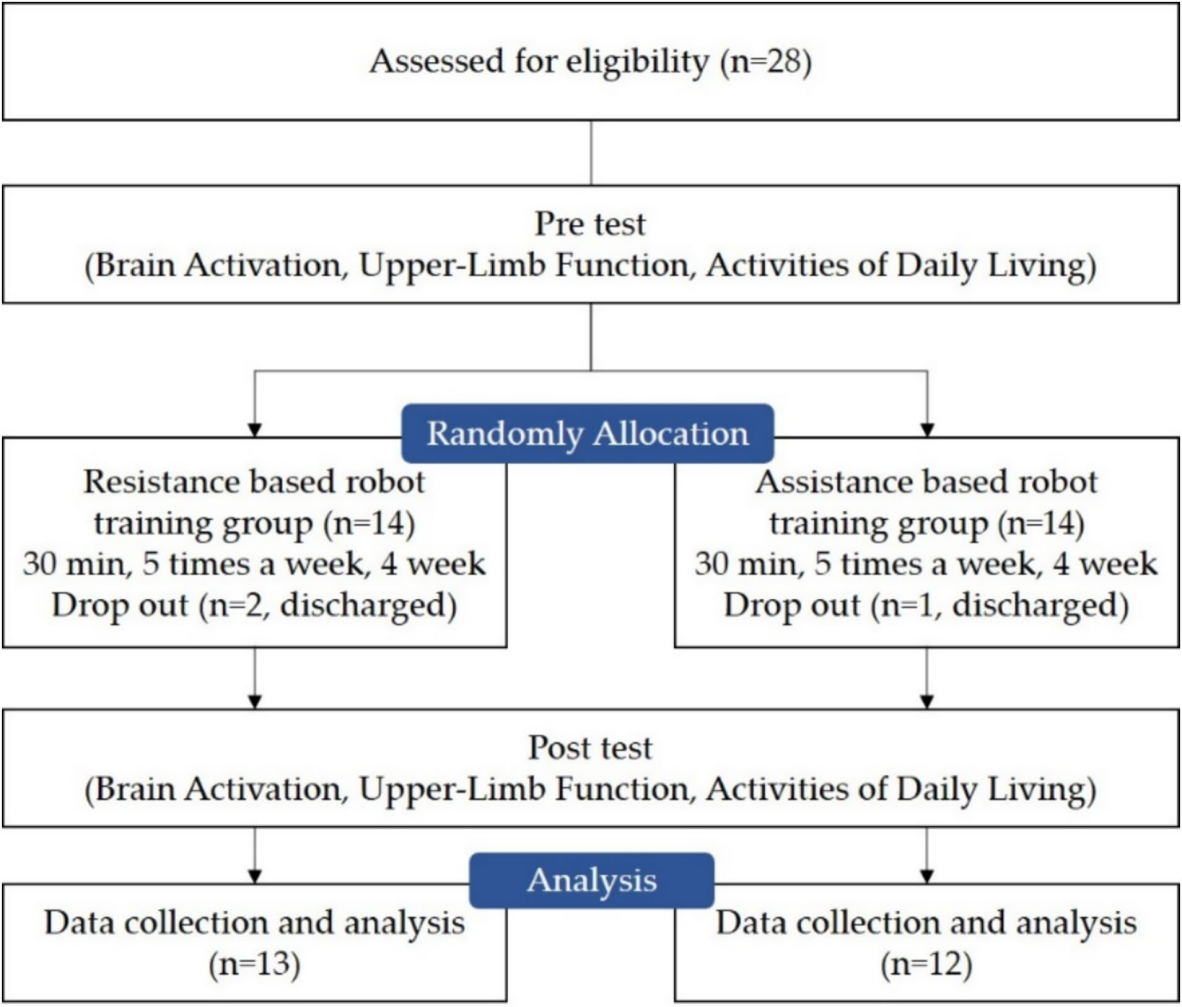
Flow chart of this study.

This study was designed as a randomized controlled trial conducted at the National Rehabilitation Center in Korea. Participants were assessed at baseline and post-intervention. Outcome measures included brain activation, upper-limb function, and activities of daily living (ADL). Brain activation was measured using functional near-infrared spectroscopy (fNIRS; NIRSIT), upper-limb function was evaluated with the Fugl-Meyer Assessment for the Upper Limb (FM-UL) and upper-limb kinematic data obtained from the InMotion 2.0 device, and ADL performance was assessed with the Motor Activity Log (MAL), which evaluates both the amount of use and quality of movement. Outcome assessments were performed by assessors who were aware of participants’ group allocation; therefore, assessor blinding was not implemented. Using a computer-generated randomization sequence, 28 participants were divided into two groups: the resistance-based robot training group (RTG, *n* = 14) and the assistance-based robot training group (ATG, *n* = 14). Three participants were excluded during the study due to discharge (RTG: *n* = 1, ATG: *n* = 2; Figure 1).

### 2.2 Participants

Participants were recruited from inpatients and outpatients at a national hospital in Seoul, Republic of Korea. Eligible participants were adults aged 19 years or older, diagnosed with first-ever hemiparetic stroke due to either cerebral infarction or cerebral hemorrhage. Inclusion criteria were: (1) no limitations in passive range of motion in the shoulder or elbow on the affected side; (2) FM-UL scores between 26 and 50; (3) sufficient cognitive ability to understand and follow instructions; (4) time since stroke onset ≥ 6 months; and (5) provision of informed consent by the patient or caregiver after a full explanation of the study. Exclusion criteria included: orthopedic or musculoskeletal disorders in the affected upper limb, cybersickness, presence of a pacemaker or other implanted electrical devices, and current pregnancy or suspected pregnancy. All participants were right-handed. As the main aim of the present study was to compare patterns of change between intervention modalities, handedness was documented descriptively and was not incorporated into the primary analyses. Because all participants were right-handed, right hemiparesis corresponded to dominant-hand involvement. The distribution of right hemiparesis did not differ between groups (RTG 6/13 vs. ATG 7/12; Table 1); therefore, dominant-hand involvement was not included as a covariate.

**Table 1:** General characteristics.

Variable	RT group (*n* = 13)	AT group (*n* = 12)	*p*
Sex (Male/Female)	10/3	9/3	0.910^a^
Paretic side (Lt/Rt)	7/6	5/7	0.543^a^
Dx (ICH/CI)	9/4	8/4	0.891^a^
Disease duration (months)	22.69 ± 12.37	21.50 ± 13.15	0.817^b^
Age (years)	57.15 ± 12.56	50.08 ± 16.68	0.241^b^
Height (cm)	163.53 ± 8.80	169.00 ± 7.21	0.105^b^
Weight (kg)	66.80 ± 9.50	68.50 ± 8.42	0.643^b^

All values are showed Mean ± SD; RT, Resistance based robot Training; AT, Assistance based robot Training; Dx, Diagnosis, ICH, Intracerebral hemorrhage, CI, Cerebral infarction, Lt, Left, Rt, Right.

a Chi-square test between two groups.

b Independent t-test between two groups.

This study was conducted in accordance with the Declaration of Helsinki, and the protocol was approved by the Ethics Committee of the National Rehabilitation Center, Korea (NRC-2023 − 05 − 034) and registered at CRIS (KCT0011076). The required sample size was calculated using G*Power 3.1.9.2, based on data from a previous study (27). With an alpha level of 0.05, an effect size of 0.25, and a power of 0.80, the minimum sample size was determined to be 24. Considering a potential dropout rate of 20%, 28 participants were recruited.

### 2.3 Primary outcome measures

#### 2.3.1 Brain activation

Brain activation was evaluated using fNIRS with the NIRSIT device (OBELAB, Republic of Korea; approved medical device). In the present study, brain activation was assessed in prefrontal regions rather than primary motor areas. This decision was based on the role of the prefrontal cortex in motor planning, executive control, and goal-directed behavior, which are critically involved in complex, resistance-based upper-limb tasks (29, 30). In addition, due to anatomical and technical constraints inherent to fNIRS—such as hair interference and reduced signal reliability over central motor regions—prefrontal areas are commonly targeted to ensure stable and reproducible measurements of task-evoked hemodynamic responses (32). Accordingly, the present fNIRS measures were intended to capture changes in task-related cognitive–motor control rather than direct motor execution. NIRSIT is a wireless system equipped with 24 laser sources (780/850 nm, maximum output <1 mW) and 32 photodetectors with source-detector distances of 1.5, 2.12, 3, and 3.5 cm, generating 204 measurement channels to construct 3D tomographic maps of the human cortex (34). The device specifications were as follows: 216 × 190 × 78 mm, 550 g, 3.7 V battery, 8 h battery life, 8.13 Hz sampling rate, maximum measurable power 2.5 μW, and wireless range up to 7 m. Detected near-infrared signals were converted into relative concentration changes of oxy-hemoglobin (O_2_Hb) and deoxy-hemoglobin (HHb) using the modified Beer–Lambert law (MBLL) (35).

**Figure 2:**
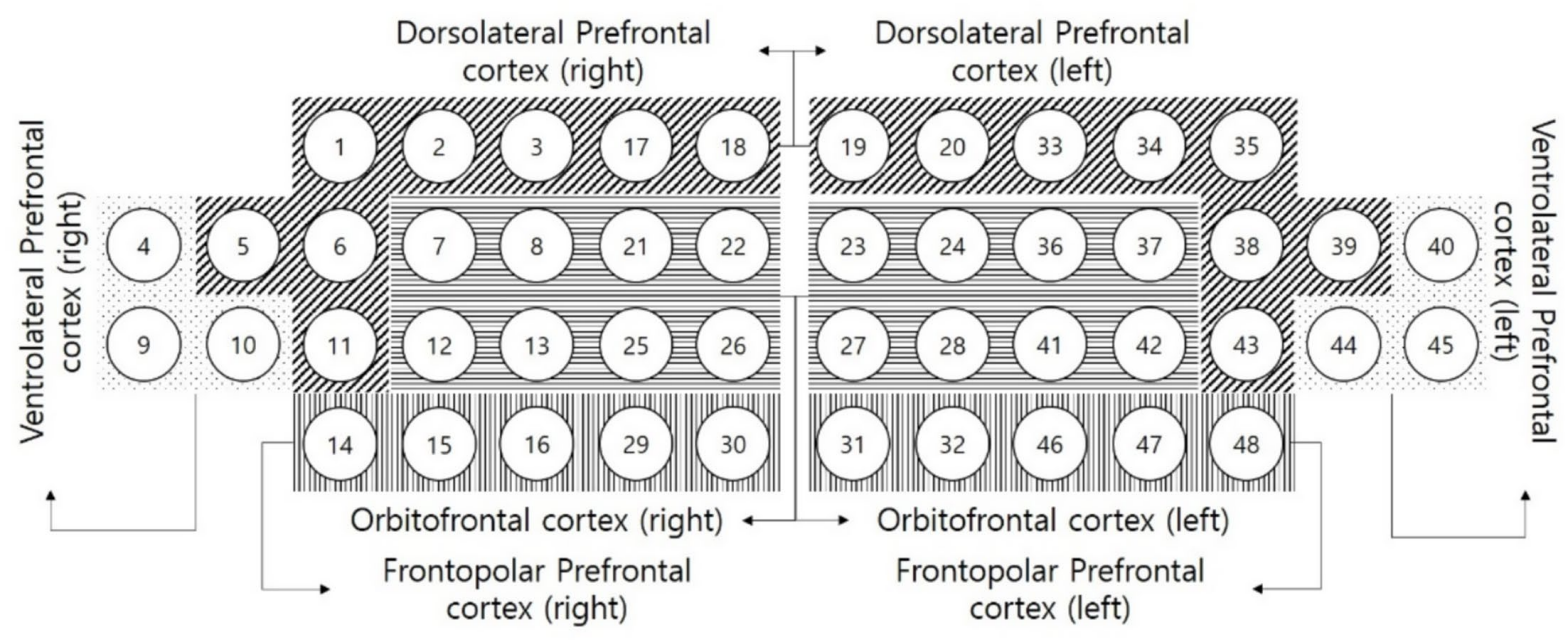
Prefrontal cortex subregions.

Mean values (± standard deviation) of O_2_Hb were collected from eight regions of interest (Figure 2) of interest: the right and left dorsolateral prefrontal cortex (DLPFC), frontopolar prefrontal cortex (FPC), ventrolateral prefrontal cortex (VLPFC), and orbitofrontal cortex (OFC). Channel distributions were as follows: DLPFC (right: 1, 2, 3, 5, 6, 11, 17, 18; left: 19, 20, 33, 34, 35, 38, 39, 43), FPC (right: 7, 8, 12, 13, 21, 22, 25, 26; left: 23, 24, 27, 28, 36, 37, 41, 42), VLPFC (right: 4, 9, 10; left: 40, 44, 45), OFC (right: 14, 15, 16, 29, 30; left: 31, 32, 46, 47, 48) (36). Based on these data, we extracted hemoglobin differences (O_2_Hb–HHb) and O_2_Hb concentration changes within the ipsilesional and contralesional hemispheres (37). Measurements were obtained during a 2-min resting baseline and throughout the InMotion 2.0 upper-limb functional assessment (38).

### 2.4 Secondary outcome measures

#### 2.4.1 Upper-limb function

##### 2.4.1.1 Fugl-Meyer assessment for the upper limb (FM-UL)

The FM-UL is part of the Fugl-Meyer Assessment, a standardized instrument developed to assess sensorimotor impairment in post-stroke patients, based on the Brunnstrom stages of motor recovery (39). It is among the most widely used quantitative measures of post-stroke motor impairment (40), and is extensively applied to plan and evaluate treatment outcomes (41). The evaluation consists of 33 items categorized into four domains: shoulder/elbow, wrist, hand, and coordination/speed. In this study, we focused on the total score (maximum = 66). The FM-UL was selected because it is widely used in clinical research on stroke rehabilitation, as it assesses both reflex activity and voluntary movements performed within synergistic patterns (42). The FM-UL demonstrates high intra- and inter-rater reliability, supporting its validity as a consistent tool for stroke assessment (43–46). Inter-rater reliability has been reported as 0.995, with moderate to high validity shown through correlations with the Motricity Index (MI) and Motor Assessment Scale (MAS; *r* = 0.639–0.891 and *r* = 0.339–0.555, respectively) (47).

##### 2.4.1.2 Upper-limb kinematic data (InMotion 2.0)

The InMotion 2.0 system provides interactive upper-limb motor training while simultaneously capturing kinematic data. The device is designed with two degrees of freedom to facilitate shoulder and elbow movements in the horizontal plane and it also functions as an assessment tool for quantifying kinematic performance. The kinematic parameters extracted from the device included movement smoothness, reach error, mean and maximum velocity, path deviation, circle size, and movement independence (48, 49).

##### 2.4.1.3 Activities of daily living

ADL performance was assessed using the Motor Activity Log (MAL), a structured interview-based questionnaire developed to evaluate the amount and quality of paretic arm use in daily life activities after stroke. The Motor Activity Log (MAL) is a semi-structured interview used to assess how often (quantity) and how well (quality) stroke patients use their affected arm in daily activities. It uses a 0–5 rating scale and is widely applied to quantify real-world arm use and evaluate rehabilitation outcomes (50).

### 2.5 Intervention

**Figure 3:**
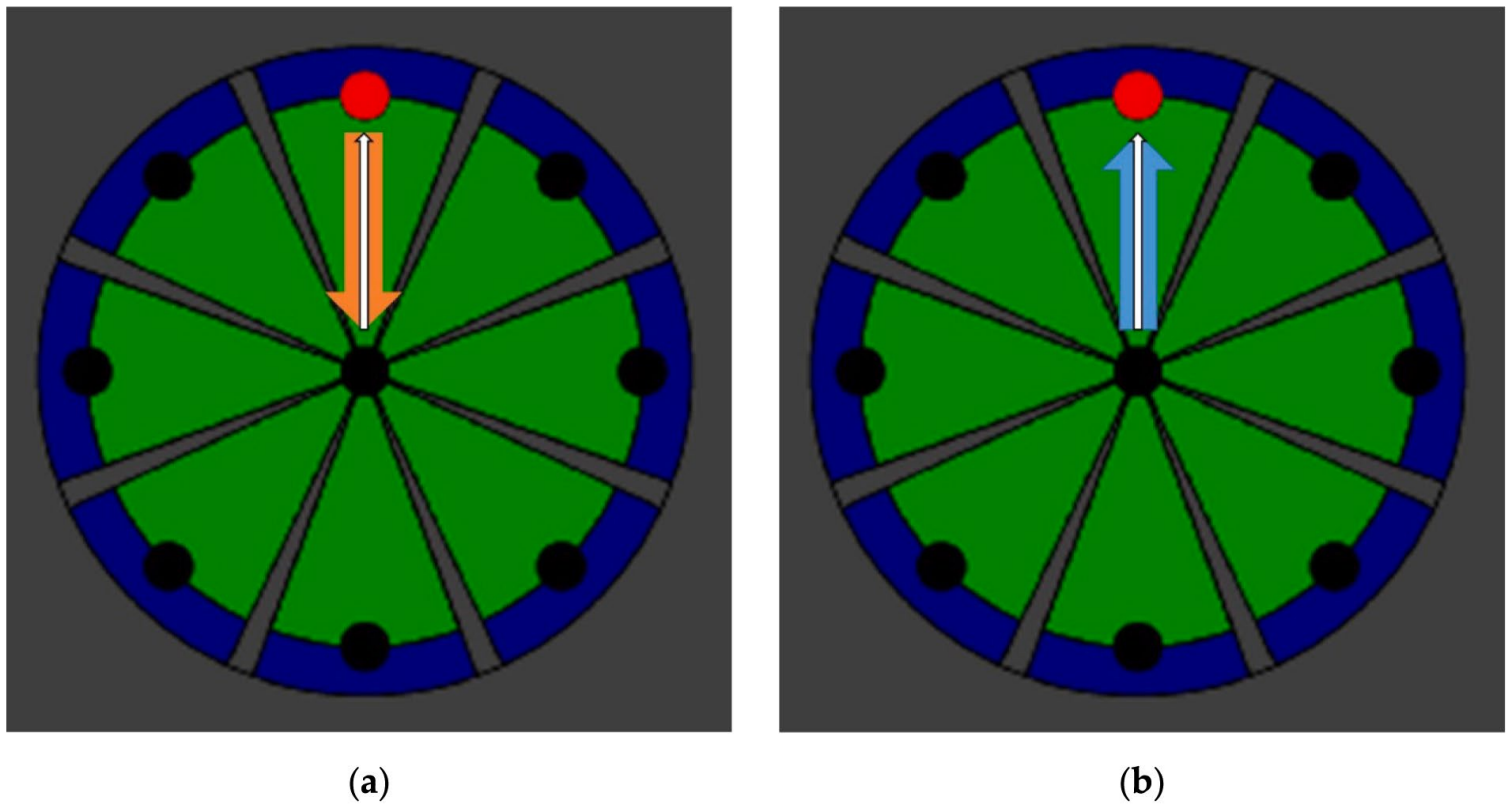
The methods of intervention using InMotion 2.0. **(a)** Resistance based robot training; **(b)** Assistance based robot training.

The intervention utilized InMotion 2.0 (Interactive Motion Technologies, Watertown, MA, United States). The device allows training and assessment of shoulder and elbow movements in the horizontal plane using an end-effector design with two degrees of freedom. Participants, seated in a chair, performed goal-directed reaching movements with their affected arm toward eight targets displayed on a screen for 30 min per session. In the RTG, participants performed movements against resistive forces opposing the target direction (Figure 3a), whereas in the ATG, movements were assisted by forces aligned with the target direction (Figure 3b), with the movement, resistive, and assisting forces represented by white, orange, and blue arrows, respectively. The intervention was delivered for 4 weeks, five sessions per week, one session daily, each lasting 30 min, Monday to Friday. Each 30-min training session consisted of point-to-point reaching movements, during which participants moved the end-effector between a central target and eight peripheral targets arranged on a 0.14-m radius circumference. Visual feedback was displayed on a monitor positioned in front of the participant. In addition to robotic training, participants received conventional physiotherapy based on standardized stroke rehabilitation protocols, including assisted stretching, shoulder and arm exercises, and functional reaching tasks.

### 2.6 Statistical analysis

Data were analyzed using SPSS version 25.0 (SPSS Inc., Chicago, IL, United States). Descriptive statistics were used to summarize the general characteristics of participants, expressed as means and standard deviations. Normality of the data was assessed using the Shapiro–Wilk test prior to statistical analysis. Baseline homogeneity between groups was assessed using the chi-square test and the independent sample t-test. Within-group changes were analyzed using paired-sample t-tests, and between-group differences in pre–post change scores were analyzed using independent-sample t-tests. Effect sizes (Cohen’s d) were calculated for pre–post change scores. Change scores were computed as the pre-intervention value minus the post-intervention value (pre–post). For within-group pre–post changes, effect sizes were calculated as the mean change divided by the standard deviation (SD) of the change scores. For between-group differences in change scores, Cohen’s d was calculated as the absolute difference between group mean change scores divided by the average SD of the change scores (SD_av = √[(SD_RT^2^ + SD_AT^2^)/2]). In a two-group pre–post design, comparing between-group differences in change scores is equivalent to testing the Group × Time interaction. A significance level of *p* < 0.05 was considered statistically significant for all analyses.

## 3 Results

### 3.1 General characteristics of the participants

A total of 28 participants were enrolled in this study, all of whom met the inclusion criteria and provided informed consent. Fourteen participants were randomly assigned to the RTG and 14 to the ATG. Three participants withdrew during the intervention period due to discharge. In the RTG, there were 10 males and 3 females, with nine cases of cerebral hemorrhage and four cases of cerebral infarction. Seven participants had left-sided hemiparesis and six had right-sided hemiparesis. The mean age was 57.15 ± 12.56 years, mean height was 163.53 ± 8.80 cm, mean weight was 66.80 ± 9.50 kg, and mean time since stroke onset was 22.69 ± 12.37 months. In the ATG, there were 9 males and 3 females, with eight cases of cerebral hemorrhage and four cases of cerebral infarction. Five participants had left-sided hemiparesis and seven had right-sided hemiparesis. The mean age was 50.08 ± 16.68 years, mean height was 169.00 ± 7.21 cm, mean weight was 68.50 ± 8.42 kg, and mean time since stroke onset was 21.50 ± 13.15 months. As all variables met the assumption of normality (*p* > 0.05), parametric tests were applied. No significant differences were observed between the two groups in their baseline characteristics (Table 1).

### 3.2 Primary outcomes

#### 3.2.1 Brain activation

##### 3.2.1.1 Ipsilesional hemispheres

Significant pre–post differences were found in all examined regions (DLPFC, VLPFC, FPC, and OFC) in both groups (*p* < 0.05). Moreover, significant between-group differences in pre–post change scores were observed in all regions (*p* < 0.05; Table 2).

**Table 2:** Brain activation (ipsilesional hemispheres) outcome measures.

Variable		RT group (*n* = 13)	AT group (*n* = 12)	t(p)/d
DLPFC (μM)	Pre	0.2851 ± 0.0215	0.2759 ± 0.0105	1.375(0.186)
	Post	0.2698 ± 0.0284	0.2714 ± 0.0135	−0.176(0.862)
	Pre-post	0.0153 ± 0.0094	0.0045 ± 0.0043	3.642(0.001)* / 1.478
	t(p), d	5.894(0.000)*, 1.634	3.673(0.004)*, 1.060	
VLPFC (μM)	Pre	0.2893 ± 0.0178	0.2858 ± 0.0127	0.576(0.570)
	Post	0.2725 ± 0.0167	0.2773 ± 0.0102	−0.872(0.392)
	Pre-post	0.0169 ± 0.0103	0.0084 ± 0.0061	2.464(0.022)* / 0.996
	t(p)	5.904(0.000)*	4.756(0.001)*	
FPC (μM)	Pre	0.2495 ± 0.0473	0.2254 ± 0.0490	1.254(0.223)
	Post	0.2322 ± 0.0565	0.2193 ± 0.0482	0.613(0.546)
	Pre-post	0.0173 ± 0.0161	0.0061 ± 0.0045	2.405(0.031)* / 0.946
	t(p)	3.861(0.002)*	4.680(0.001)*	
OFC (μM)	Pre	0.2809 ± 0.0091	0.2739 ± 0.0166	1.318(0.200)
	Post	0.2698 ± 0.0122	0.2690 ± 0.0163	0.133(0.896)
	Pre-post	0.0111 ± 0.0061	0.0049 ± 0.0031	3.166(0.004)* / 1.284
	t(p)	6.518(0.000)*	5.508(0.000)*	

All values are presented as Mean ± SD; RT, Resistance based robot Training; AT, Assistance based robot Training; DLPFC, dorsolateral prefrontal cortex; VLPFC, ventrolateral prefrontal cortex; FPC, frontopolar prefrontal cortex; OFC, orbitofrontal cortex; *p < 0.05. d: Cohen’s d effect size for between-group differences in pre–post change scores (change = pre − post), computed as |Δmean|/SD_av, where SD_av = √[(SD_RT^2^ + SD_AT^2^)/2].

##### 3.2.1.2 Contralesional hemispheres

Significant pre–post differences were observed in the dorsolateral prefrontal cortex (DLPFC), ventrolateral prefrontal cortex (VLPFC), and frontopolar cortex (FPC) in both groups (*p* < 0.05). However, no significant between-group differences were identified in the change scores. In the orbitofrontal cortex (OFC), significant pre–post differences were found only in the RTG (Table 3).

**Table 3:** Brain activation (contralesional hemispheres) outcome measures.

Variable		RT group (*n* = 13)	AT group (*n* = 12)	t(p)/d
DLPFC (μM)	Pre	0.1862 ± 0.0105	0.1804 ± 0.0093	1.469(0.155)
	Post	0.1762 ± 0.0103	0.1749 ± 0.0064	0.369(0.716)
	Pre-post	0.0100 ± 0.0067	0.0055 ± 0.0048	1.936(0.065) / 0.780
	t(p)	5.360(0.000)*	3.954(0.002)*	
VLPFC (μM)	Pre	0.1780 ± 0.0101	0.1728 ± 0.0092	1.357(0.188)
	Post	0.1708 ± 0.0148	0.1660 ± 0.0115	0.908(0.373)
	Pre-post	0.0072 ± 0.0093	0.0068 ± 0.0032	0.152(0.881) / 0.059
	t(p)	2.784(0.017)*	7.297(0.000)*	
FPC (μM)	Pre	0.1705 ± 0.0152	0.1622 ± 0.0111	1.549(0.135)
	Post	0.1605 ± 0.0191	0.1547 ± 0.0127	0.879(0.389)
	Pre-post	0.0100 ± 0.0104	0.0075 ± 0.0062	0.735(0.469) / 0.297
	t(p)	3.490(0.004)*	4.180(0.002)*	
OFC (μM)	Pre	0.0995 ± 0.0473	0.0662 ± 0.0558	1.614(0.120)
	Post	0.0853 ± 0.0594	0.0604 ± 0.0565	1.070(0.296)
	Pre-post	0.0142 ± 0.0170	0.0057 ± 0.0108	1.469(0.155) / 0.593
	t(p)	3.004(0.011)*	1.844(0.092)	

All values are presented as Mean ± SD; RT, Resistance based robot Training; AT, Assistance based robot Training; DLPFC, dorsolateral prefrontal cortex; VLPFC, ventrolateral prefrontal cortex; FPC, frontopolar prefrontal cortex; OFC, orbitofrontal cortex; *p < 0.05. d: Cohen’s d effect size for between-group differences in pre–post change scores (change = pre − post), computed as |Δmean|/SD_av, where SD_av = √[(SD_RT^2^ + SD_AT^2^)/2].

### 3.3 Secondary outcomes

#### 3.3.1 Upper-limb function

##### 3.3.1.1 Fugl-Meyer assessment for the upper limb (FM-UL)

Both groups showed significant pre–post improvements in FM-UL scores (*p* < 0.05). However, there were no significant between-group differences in change scores (Table 4).

**Table 4:** Upper-limb function outcome measures.

Variable		RT group (*n* = 13)	AT group (*n* = 12)	t(p)/d
FM-UL (score)	Pre	38.23 ± 8.17	38.91 ± 7.85	−0.214(0.833)
	Post	41.46 ± 7.34	40.66 ± 7.72	0.264(0.794)
	Pre-post	−3.23 ± 3.24	−1.75 ± 2.13	−1.335(0.195) / 0.539
	t(p)	−3.590(0.004)*	−2.836(0.016)*	
UL-KD smoothness	Pre	0.4821 ± 0.0589	0.5027 ± 0.0516	−0.927(0.364)
	Post	0.5299 ± 0.0502	0.5273 ± 0.0466	0.138(0.892)
	Pre-post	−0.0478 ± 0.0333	−0.0246 ± 0.0150	−2.213(0.037)* / 0.898
	t(p)	−5.167(0.000)*	−5.673(0.000)*	
UL-KD mean velocity (m/s)	Pre	0.0804 ± 0.0327	0.0785 ± 0.0284	0.157(0.876)
	Post	0.0983 ± 0.0290	0.0952 ± 0.0282	0.274(0.787)
	Pre-post	−0.0179 ± 0.0188	−0.0167 ± 0.0388	−0.100(0.921) / 0.039
	t(p)	−3.441(0.005)*	−1.491(0.164)	
UL-KD max velocity (m/s)	Pre	0.1897 ± 0.0697	0.1738 ± 0.0408	0.687(0.499)
	Post	0.2022 ± 0.0704	0.1766 ± 0.0427	1.086(0.289)
	Pre-post	−0.0125 ± 0.0406	−0.0028 ± 0.0169	−0.792(0.440) / 0.312
	t(p)	−1.108(0.290)	−0.563(0.585)	
UL-KD reach error (cm)	Pre	0.0152 ± 0.0134	0.0100 ± 0.0057	1.266(0.223)
	Post	0.0102 ± 0.0043	0.0087 ± 0.0012	1.164(0.256)
	Pre-post	0.0050 ± 0.0139	0.0013 ± 0.0046	0.896(0.385) / 0.352
	t(p)	1.293(0.220)	0.996(0.341)	
UL-KD path error (cm)	Pre	0.0134 ± 0.0067	0.0101 ± 0.0032	1.590(0.130)
	Post	0.0118 ± 0.0056	0.0098 ± 0.0034	1.083(0.290)
	Pre-post	0.0016 ± 0.0057	0.0003 ± 0.0015	0.777(0.450) / 0.305
	t(p)	1.015(0.330)	0.771(0.457)	
UL-KD circle size (m)	Pre	0.0162 ± 0.0026	0.0175 ± 0.0025	−1.258(0.221)
	Post	0.0172 ± 0.0017	0.0177 ± 0.0021	−0.661(0.515)
	Pre-post	−0.0010 ± 0.0011	−0.0002 ± 0.0015	−1.457(0.159) / 0.579
	t(p)	−3.224(0.007)*	−0.534(0.604)	
UL-KD circle independence	Pre	0.7872 ± 0.1004	0.7924 ± 0.0976	−0.133(0.896)
	Post	0.8437 ± 0.0754	0.8402 ± 0.0547	0.133(0.896)
	Pre-post	−0.0565 ± 0.0368	−0.0478 ± 0.0899	−0.325(0.748) / 0.127
	t(p)	−5.543(0.000)*	−1.841(0.093)	

All values are presented as Mean ± SD; RT, Resistance based robot Training; AT, Assistance based robot Training; DLPFC, dorsolateral prefrontal cortex; VLPFC, ventrolateral prefrontal cortex; FPC, frontopolar prefrontal cortex; OFC, orbitofrontal cortex; *p < 0.05. d: Cohen’s d effect size for between-group differences in pre–post change scores (change = pre − post), computed as |Δmean|/SD_av, where SD_av = √[(SD_RT^2^ + SD_AT^2^)/2].

##### 3.3.1.2 Upper-limb kinematic data (InMotion 2.0)

For smoothness, both groups demonstrated significant pre–post improvements (*p* < 0.05), and significant between-group differences were observed in the change scores (*p* < 0.05). In the RTG, significant pre–post improvements were also found in mean velocity, circle size, and independence (*p* < 0.05), whereas no significant changes were observed in the ATG for these measures (Table 4).

#### 3.3.2 Activities of daily living

For ADL performance assessed by the Motor Activity Log, both the amount of use and quality of movement showed significant pre–post improvements in both groups (*p* < 0.05). However, no significant between-group differences were observed in the change scores (Table 5).

**Table 5:** Activities of daily living outcome measures.

Variable		RT group (*n* = 13)	AT group (*n* = 12)	t(p)/d
MAL quantity (score)	Pre	11.61 ± 10.89	15.62 ± 15.12	−0.765(0.452)
	Post	13.92 ± 10.49	17.50 ± 14.64	−0.706(0.487)
	Pre-post	−2.30 ± 2.59	−1.87 ± 2.35	−0.435(0.667) / 0.173
	t(p)	−3.207(0.008)*	−2.757(0.019)*	
MAL quality (score)	Pre	6.19 ± 9.76	11.54 ± 13.83	−1.124(0.273)
	Post	8.38 ± 9.65	12.95 ± 13.25	−0.992(0.331)
	Pre-post	−2.19 ± 3.17	−1.41 ± 2.15	−0.708(0.486) / 0.287
	t(p)	−2.487(0.029)*	−2.281(0.043)*	

All values are presented as Mean ± SD; RT, Resistance based robot Training; AT, Assistance based robot Training; MAL, Motor Activity Log; *p < 0.05. d: Cohen’s d effect size for between-group differences in pre–post change scores (change = pre − post), computed as |Δmean|/SD_av, where SD_av = √[(SD_RT^2^ + SD_AT^2^)/2].

## 4 Discussion

This study compared the effects of resistance-based robot training and assistance-based robot training on brain activation, upper-limb function, and ADL in patients with stroke. The results demonstrated that brain activation significantly decreased after the intervention in most regions in both groups, with a significant between-group difference observed in the ipsilesional hemispheres. For upper-limb function, the RTG showed significant improvements in FM-UL scores, as well as in smoothness, mean velocity, and circle size in the kinematic data. In contrast, the ATG showed significant improvements only in FM-UL scores and smoothness. In terms of ADL, both groups demonstrated significant pre–post improvements in MAL scores; however, no significant between-group differences were observed.

Analysis of the prefrontal cortex activation revealed that in both groups, activity in the ipsilesional DLPFC, VLPFC, FPC, and OFC decreased significantly after the intervention. Similarly, contralesional DLPFC, VLPFC, and FPC activity decreased in both groups, while OFC activity decreased significantly only in the RTG. The prefrontal cortex is known to play a role in motor control and adaptation to changes in motor state and has been shown to correlate with motor function (51). Stroke patients with impaired motor abilities generally require greater attentional resources, resulting in higher prefrontal activation during movement (51). However, the observed reduction in activation after the intervention may reflect reduced cognitive/attentional demand or task familiarity during the assessment rather than neural efficiency per se, particularly because FM-UL improved similarly in both groups without significant between-group differences. Moreover, the prefrontal cortex is activated during physically demanding tasks and when sustained attention is required (52, 53). The greater reduction in activation in the RTG compared to the ATG suggests that resistance-based movements, which impose higher task demands, initially required stronger activation. With practice-related improvements in task performance (e.g., kinematic smoothness), prefrontal activity may decrease as executive demands were reduced (36, 54). Given that stroke patients typically exhibit reduced HbO levels in the prefrontal cortex compared with healthy individuals (51), interventions that may reduce compensatory prefrontal engagement while improving task performance are clinically relevant.

In terms of motor outcomes, FM-UL scores improved significantly in both groups, and kinematic parameters indicated enhanced upper-limb function. Previous studies have shown beneficial effects of both resistance- and assistance-based robotic therapies (28, 55). In the present study, both the RTG and ATG demonstrated significant improvements in FM-UL scores, with no significant differences in the magnitude of change between groups. The effectiveness of resistance versus assistance training likely depends on the functional status and recovery stage of stroke patients (56, 57). In this trial, participants in the chronic stage with sufficient residual ability may have benefited more from resistance training, which promoted active participation and increased voluntary engagement by reducing ‘motor slacking’. This interpretation aligns with prior research indicating that resistance can sustain patient engagement, encourage voluntary effort, and mitigate motor slacking (27, 57, 58). Therefore, resistance-based robotic therapy may be particularly effective for patients with chronic stroke who are not severely impaired. Furthermore, resistance training enhances motor control, strengthens muscles, and facilitates motor learning, thereby contributing to functional recovery (27, 59).

Regarding ADL performance, MAL scores indicated significant pre–post improvements in both groups, reflecting increased frequency and quality of paretic arm use. While some previous studies found no significant changes (60, 61), others combining robotic therapy with CIMT reported significant improvements (62, 63). The improvements observed in this study may be attributed to the focus on paretic limb rehabilitation, and although the treatment duration was shorter than that of CIMT, similar therapeutic effects were observed. Additionally, Liu’s study (42) also reported similar findings, possibly because it was conducted with inpatient populations where rehabilitation therapy was prioritized over everyday activities. As the participants in the present study were also inpatients receiving consistent rehabilitation, this may have contributed to the observed treatment effects.

Several limitations should be acknowledged. First, brain activation was assessed in prefrontal regions rather than directly in primary motor cortical areas. Accordingly, the fNIRS findings reflect task-related changes in higher-order motor planning and executive control processes rather than direct measures of motor execution. Therefore, interpretations regarding neural mechanisms should be made with appropriate caution. Because motor recovery is primarily mediated by activity in regions such as the primary motor cortex, premotor cortex, and supplementary motor area, this indirect measurement may have limited our ability to accurately interpret the neurophysiological mechanisms associated with the intervention. Second, the study assessed short-term outcomes over 4 weeks, and the persistence of treatment effects could not be confirmed due to difficulties in follow-up after discharge. Third, the lack of assessor blinding may have introduced assessment bias. Fourth, although all participants were in the chronic stage of stroke, recovery stages were not further stratified, and simple randomization was applied to focus on individuals requiring ongoing rehabilitation beyond the period of rapid spontaneous recovery. Nevertheless, heterogeneity in recovery stage may have influenced individual responsiveness to the intervention. This may have introduced imbalances in baseline motor function or recovery potential across groups, which could have influenced the comparability of treatment effects. Therefore, the results should be interpreted with caution, as differences in recovery stages may partially account for the observed outcomes. Finally, because participants were inpatients, it was not possible to fully control for concurrent treatments.

## 5 Conclusion

This study compared resistance-based and assistance-based robotic training for the upper limb in patients with stroke, focusing on prefrontal activation, motor function, and activities of daily living. Resistance-based training was associated with greater reductions in prefrontal activation within the ipsilesional hemispheres after the intervention compared with assistance-based training. Notably, the resistance group demonstrated additional improvements in upper-limb kinematic smoothness. Together, these findings suggest that resistance-based robotic training may induce distinct patterns of prefrontal cortex engagement and support kinematic aspects of motor performance in patients with chronic stroke.

## Data Availability

The raw data supporting the conclusions of this article are not publicly available due to ethical and privacy restrictions related to clinical participant data, but are available from the corresponding author upon reasonable request.
